# Editorial

**Published:** 1864-07

**Authors:** 


### Editorial.

### RECORD OF PROCEEDINGS OF THE AMERICAN MEDICAL ASSOCIATION. FIFTEENTH ANNUAL MEETING. HELD IN IRVING HALL, IN THE CITY OF NEW YORK, COMMENCING JUNE 7, 1864.

The following record has been compiled from the proof-sheets of the *New York Medical Independent*, kindly furnished us by the editors of that journal, and for which we return them our thanks.

### FIRST DAY—MORNING SESSION.

At 11 o'clock, A. M., the Association was called to order by the President, Dr. ALDEN MARCH, of Albany, N. Y., aided by Vice-Presidents JAMES COUPER, of Delaware, D. PRINCE, of Illinois, and C. C. COX, of Vol. Med. Staff of U. S. Army.

The Secretaries, Drs. H. A. JOHNSON, of Illinois, and G. FURMAN, of New York, were also present and took their seats at the table.

Rev. Dr. DEWITT opened the exercises by a very appropriate and impressive prayer.

Dr. James Anderson, Chairman of the Committee of Arrangements, then read the report of the committee, concerning the reception and registration of members and delegates, and in behalf of the committee cordially welcomed the members of the Association to the hospitalities of their brethren in the great Commercial Metropolis of the Union. He closed by suggesting that the Association should hold its meetings from 10 o'clock A.M., to 1½ o'clock P.M., and from 3 o'clock P.M., to such hour as they might choose to adjourn.

On motion, the recommendations of the report were adopted by the Association.

The junior Secretary, Dr. Furman, then read the roll of members. The President invited all the ex-Presidents present, together with Dr. Charles Tripler, Surgeon of the U.S. Army, to take seats on the platform.

On motion of Dr. Bissell, of Utica, a recess of 10 minutes was taken, to enable the delegates from each State to select one of their number to act as a member of the Committee to nominate Officers and Committees for the ensuing year. At the close of the recess, the following were reported as the names of the Nominating Committee, viz.:—J. C. Weston, of Maine, J. N. Stiles, of Vermont, T. D. Marshall, of N.H., H. R. Storer, of Mass., J. Gardner, of R.I., B. H. Catlin, of Ct., J. P. White, of N.Y., L. A. Smith, of N.J., A. Nebinger, of Pa., F. E. B. Hintze, of Md., N. Young, of D.C., B. B. Leonard, of Ohio, J. F. Hibbard, of Ind., W. H. Byford, of Illinois, S. G. Armour, of Mich., A. E. McCurdy, of Iowa, George W. Phelps, of Mo., H. F. Askew, of Del., J. K. Bartlett, of Wis., Thomas Antisell, of U.S. Army, and Thomas L. Smith, of U.S. Navy.

The President, Dr. Alden March, then read his Annual Address.

He briefly reviewed the history of the Association, more especially in reference to its connection with the subject of Medical Education; paid a deserved tribute to his predecessors in the Presidential Chair; and urged a steady continuous effort to still further improve the educational interests of the whole profession.

The address was listened to with interest and pleasure, and an unanimous vote of thanks tendered to the author, with a request that a copy be furnished to the Committee of Publication.

The Association then adjourned until 3 o'clock P.M.

AFTERNOON SESSION.

At 3 o'clock P.M., the Association was called to order by the President, and the Secretary read the minutes of the morning session, which were approved.

Dr. Anderson, from the Committee of Arrangements, reported the names of additional delegates, and announced the following evening entertainments, viz.:—On Tuesday evening, at 8 o'clock, by Hon. C. Godfrey Gunther, Mayor of the City, and Drs. Joseph M. Smith, Charles A. Budd, Isaac E. Taylor, and Gurdon Buck; on Wednesday evening, by Drs. Willard Parker, James Anderson, Alonzo Clark, and Jared Linsley; and on Thursday evening, by Dr. E. R. Squibb, at his Laboratory in Brooklyn. Friday was set apart for an excursion from Bellvue Hospital to the Institutions on Randall's and Blackwell's Islands.

Dr. S. G. Armour, Chairman of the committee on nominations, presented the following report: For President, Dr. N. S. Davis, of Illinois; Vice Presidents, Drs. Wm. H. Mussey, of Ohio, Worthington Hooker, of Connecticut, Wm. Wheeler, of D.C., and F. E. B. Hinsey, of Maryland; Treasurer, Dr. Casper Wistar, of Pennsylvania; Secretary, Dr. G. Furman, of New York. The committee also recommended Boston as the next place of meeting.

Dr. Griscom, of New York, moved that the report be accepted and laid on the table, for the purpose of acting on an amendment to the Constitution relating to the term of office of the President and Vice-Presidents, which was proposed at the last annual meeting. After some discussion the motion of Dr. Griscom was lost.

Dr. Raphael, of New York, moved that the report be recommitted to the committee, with instructions to report the names of two or more candidates for the office of President, from which the Association should choose by ballot. This led to a discussion in which Drs. Raphael, Currie, Corliss, Cohen, Bissell, Kennedy, Ramsey, and Jewell took part. But the motion to re-commit was lost. The question was then taken on the adoption of the report of the committee on nominations, and carried almost unanimously. The President appointed Drs. Bissell, of New York, and Askew, of Delaware, a committee to escort the newly elected officers to the chair.

The President elect, Dr. N. S. Davis, of Chicago, Illinois, on being conducted to the chair, expressed his gratitude for the honor conferred upon him—alluded to his identification with the Association, from the initiatory steps to its organization to the present time—declared his unabated attachment to the profession—and alluded regretfully to the continued absence of delegates from the South. The speech was brief, terse, conciliatory, and closed with no other allusion to the rebellion than the expression of an earnest hope, that we should soon again have the pleasure of greeting our Southern brethren at our annual re-unions, together with a full restoration to the glorious old union, of every foot of territory they represent.

Drs. A. B. Palmer, H. F. Askew, and S. G. Hubbard, were appointed by the chair a special committee to receive and examine all volunteer papers, designed to be presented to the Association.

On motion, the regular order of business was suspended for the purpose of acting on an amendment to the Constitution proposed at the last meeting, in relation to the appointment of a permanent secretary.

The proposed amendments, providing for the appointment of one *permanent* and one assistent Secretary, and defining their duties, having been fully stated by the chair, and discussed by Drs. Wistar, Gardner, Hooker, Kennedy, Mauran, Loomis, Bond, and Jewell, they were unanimously adopted.

The Association than adjourned until 10 o'clock A. M.

SECOND DAY—MORNING SESSION.

At 10 o'clock A. M., the Association was called to order, the President, Dr. N. S. Davis, being in the chair. The minutes of the preceding meeting were read by Secretary Furman, and approved. Several gentlemen were elected members by invitation. Dr. Brown-Séquard, of Boston, and Dr. John P. Gray, of the Lunatic Asylum, at Utica, were elected permanent members. The former, on being invited to take a seat on the platform, made a brief and modest acknowledgement of the honor conferred upon him. The regular order of business being the reports of officers and standing committees, the whole list was called in order, and the several reports referred to the appropriate sections for consideration.

Resolutions concerning the relative rank and pay of army Surgeons, were offered by Dr. C. C. Cox, and after some discussion by Drs. Cox, Hamilton, of N. Y., and Mussey, of Cincinnatti, they were adopted.

On motion, a vote of thanks was unanimously tendered to Dr. Alden March, the retiring President of the Association. At 1½ o'clock P. M., the Association adjourned, to meet in sections in the afternoon.

At 3 o'clock P. M., the sections on Chemistry, and Materia Medica; Practical Medicine and Obstetrics; Surgery; and on Meteorology, Medical Topography, Epidemic Diseases, &c., were organized, and proceeded to the hearing and discussion of the several reports and papers referred to them.

THIRD DAY—MORNING SESSION.

The Convention re-assembled this morning, Dr. N. S. Davis, of Chicago, in the Chair, and Dr. Guido Furman, of N. York, Secretary.

After reading the minutes, Dr. Childs, of Mass., on invitation, took a seat on the platform, and thanked the Association for the honor extended to him.

Dr. Mauran, of R. I., called the attention of the Convention to the prizes offered by the Rhode Island State Medical Society, amounting to $200, for the best essay on the following subjects:

1. What evidence is there that Inflammatory and Febrile Diseases have undergone any general change of type?
2. The effects of climate in America on Tuberculous Diseases.

On motion of Dr. Cox, Dr. Charles S. Tripler, U. S. A., was made Chairman of the Committee on memorializing Congress for better assimilation of rank and pay among army surgeons.

A letter was received from Dr. R. Hills, of the Central Ohio Lunatic Asylum, Chairman of Committee on Insanity, in which he requests a continuance of the Committee, the report not being completed. The request was granted, and on motion of Dr. J. H. Griscom, of N. Y., Dr. E. H. Van Duzen, of Mich., was added to the committee.

Dr. Butler, of Phila., Secretary of the Section on Meteorology, Medical Topography, Epidemic Diseases, and Medical Jurisprudence and Hygiene, reported a series of resolutions on compulsory vaccination, favoring the enactment of laws in the different States to this purpose.

The same Section reported back for publication the communication of Dr. Griscom of this city, on "Physiological and Dietetic relations of Phosphorus;" also the paper presented by Dr. Cyrus Ramsey, on Sanitary Science and Mortuary Statistics of New York, from 1850 to 1864.

Dr. E. R. Squibb, of this city, from Committee on the Practical Workings of the U. S. Law relating to the Inspection of Drugs and Medicines, presented a report. The report goes back to the origin of the Drug Law in 1848, and the workings of it since. The fixing of the standard was liberal, so as not to impede the drug trade. So, for instance, was Jalap admitted with 11 per cent. Jalap Resin, while good Jalap contained 12 to 14 per cent. resinous matter, and Scammony with 73 per cent.; Scammony, white and good article always contains 75 per cent. Political influence, however, had debased the execution of the law, so as to make it a dead letter on the statute-book. Inferior Cinchona bark, Jalap with resin extracted, Senna is rarely met with in a pure state, and is generally found much adulterated with sticks and stones. The whole mal-administration of the law is the result of bad appointments by the Secretary of the Treasury. So, for instance, had Mr. Chase made the appointment of Examiners in direct opposition to the requests of the State Medical Societies, aided by the Academy of Medicine, College of Physicians, &c. These societies had suggested an examination of the applicants by the Medical Boards of the Army and Navy, and that they be graduates of the Medical Colleges. The same request was made by the leading drug houses in the country. All the principal ports of entry were represented in the movement. The Committee who presented the petitions were promised that they would be heard, and their report receive attention. But they were ignored, and men appointed totally unfit for the position, for at no time, were there so many spurious and bad drugs in the market as within the last year or two. The parties to be blamed for this were first, the Secretary of the Treasury, and next the incumbents of the office, who break their oath of office frequently. The report recommends some action on this state of affairs, as soon the time would come when new appointments would be made.

Dr. Percy moved that the report be laid on the table, as it assailed the officers of the Government, but it was lost by a vote of the whole house but two.

After discussion by Drs. Percy, Squibb, Loomis, Jewell, Currie, and McFarland, the paper of Dr. Squibb was referred to the Section on Chemistry and Materia Medica.

The Secretary of the Section on Practical Medicine, Dr. Storer, of Boston, reported that the paper of Dr. Gardner, of N. Y., on the use and abuse of Pessaries, had been referred to the Committee on Publication, as was also the paper presented by Dr. Leving, of Pa., on Spotted Fever. Dr. Storer also read a paper on the "Relations of Female Patients to Hospitals for the Insane; the necessity, on their account, of a Board of Consulting Physicians to every hospital;" which was also referred to the Committee on Publication, and referred resolutions carrying out this view to the full Association for confirmation.

The resolutions of Dr. Storer read as follows:

*Resolved*, That in the opinion of the American Medical Association, it is expedient that there should be attached to every public hospital for the insane, one or more consulting physicians [whose appointment should be honorary], who may be consulted at the discretion of the Superintendent; such measure being for the interest of the hospital, its medical officers, and its patients.

*Resolved*, That a copy of the above resolution be transmitted to the Board of Trustees of each of our public hospitals for the insane, and also to the Secretary of the Association of American Superintendents, with the request that it may be endorsed by that body, and the act proposed to be urged upon the respective boards with which the members are officially connected.

These resolutions were discussed by Drs. Griscom, Storer, Gardner, McArthur, and Bronson. On motion of Dr. Griscom the words inclosed in brackets in the first resolution were stricken out, and as thus amended the resolutions were adopted.

Dr. Griscom spoke at some length on the appointment of a Permanent Secretary, as requiring a person of eminent qualifications. He offered a resolution to the effect that the Secretary be entitled to a compensation from the surplus funds of the Society, after all claims for each current year shall have been paid.

Dr. Kennedy, of New York, moved that the salary be fixed at $1,500, which was amended by Dr. Sayre, by making it $5,000.

Dr. Raphael, of New York, was opposed to the salary, as experience here taught that no such sums were ever left over.

The Chair called the attention of the meeting to the clause in the Constitution fixing the compensation of the Secretary to his expenses in attending the Convention, and for maintaining the correspondence. He therefore should rule the question out of order.

Dr. Griscom appealed from the decision of the Chair, which appeal he afterwards withdrew.

Dr. Kennedy moved a reconsideration of the resolution adopted yesterday on the Constitution.

Dr. L. A. Sayre moved that the members tax themselves sufficiently to pay a salary of $1,500 to the Secretary.

Dr. Palmer, of Michigan, advised to lay further discussion over. The matter dropped here.

The Committee on Nominations, Dr. Armour, of Michigan, reported the following Standing Committees:

*On Insanity.*—Ralph Hills, Ohio; C. H. Nichols, Dist. of Col.; D. P. Bissell, N. Y.; S. W. Butler, Pa.; John S. Butler, Ct.; E. R. Van Duzen, Mich.

*On Exsection and its Connection with Conservative Surgery.*—Lewis A. Sayre, N. Y.; G. W. Norris, Pa.; G. C. Blackman, Ohio; H. S. Tewksberry, Me.; E. Andrews, Ill.; G. B. Twitchell, N. H.; G. C. Hughes, Iowa; Geo. Clymer, U. S. N.; J. R. W. Dunbar, Md.; R. H. Gilbert, U. S. A.

*On Alcohol and its Relation to Man.*—G. E. Morgan, Md.

*On Microscopic Observations on Cancer Cells.*—L. J. Sanford, Ct.

*On Medical Ethics.*—J. J. Murphy, Ohio; M. L. Linton, Mo.; B. F. Schneck, Pa.; S. Wickersham, Ill.; A. J. Fuller, Me.

*On the Microscope.*—J. M. Corse, Pa.

*On Quarantine.*—D. D. Clark, Pa.; E. M. Snow, R. I.; W. Jewell, Pa.; E. D. Fenner, La.; J. W. Houck, Md.

*On Causes of the Extinction of the Aboriginal Races of America.*—G. Suckley, N. Y.

*On the Relations which Electricity sustains to the Cause of Disease.*—S. Littell, Pa.

*On the Morbid and Therapeutic Effect of Mental and Moral Influences.*—A. B. Palmer, Mich.

*On the Causes and Treatment of Ununited Fractures.*—F. H. Hamilton, N. Y.

*On Diphtheria.*—L. Clark, Ill.

*On the Drainage and Sewerage of large Cities, and their Influences on Public Health.*—W. J. C. Duhamil, Dist. of Col.; E. C. Baldwin, Md.; C. Ramsey, N. Y.

*On the Uses and Abuses of Pessaries.*—J. P. White, N. Y.

*On International Medical Ethics, to Investigate the Conditions demanded for a Diploma of Doctor of Medicine in the various Medical Schools and Universities of Europe.*—J. B. Upham, Mass.; R. Thompson, Ohio; G. C. Shattuck, Mass.; G. C. E. Weber, Ohio.

*On Climatology and Epidemic Diseases.*—C. W. Parsons, R. I.; P. A. Stackpole, N. H.; T. M. Logan, Cal.; R. C. Hamill, Ill.; J. C. Weston, Me.; B. H. Catlin, Ct.; C. S. Allen,

Vt.; T. Antisell, Dist. of Col.; J. W. H. Baker, Iowa; A. Sager, Mich.; O. S. Mahon, Md.; J. W. Russell, Ohio; D. F. Condie, Pa.; H. Townsend, N. Y.

*On Autopsies in relation to Medical Jurisprudence.*—F. C. Finnell.

*On so-called Spotted Fever.*—J. J. Levick.

*On the Introduction of Disease by Commerce, and the means for its Prevention.*—A. N. Bell, Brooklyn.

*On Patent Rights and Medicine Men.*—D. Prince, Ill.; T. Antisell, Dist. of Col.; S. Smith, N. Y.

Dr. W. B. Atkinson, of Philadelphia, was proposed for Permanent-Secretary of the Association, and Dr. Storer, of Boston, for Assistant-Secretary.

Dr. Kennedy moved the substitution of the name of Dr. Furman as Secretary, paying a high eulogium to the character of Dr. Furman.

Dr. Gardner was opposed to the nomination of a gentleman from Philadelphia, Pennsylvania having all the patronage from the Society by having the Board of Publication, the Treasurer, etc. He would move that the nomination be sent back to the Committee.

Dr. Nebinger, of Pa. defended the Committee from the charge of having favored a particular State.

The nomination of Dr. Furman was lost by a vote of 56 for 75 against, whereupon Dr. W. B. Atkinson was elected unanimously.

The Committee on Prize Essays, through Dr. Peaselee, of N. Y., reported that they had received three Essays for competition, viz: On the surgical treatment of morbid growths in the Larynx, which was received too late to be acted upon. This essay was written with excellent care, and showed great research on the subject.

What effect has the milk and meat of diseased animals on public health? The Committee gave this a high eulogium and expressed a hope for its publication.

The third essay "Pathology of Jaundice," was given the prize. Dr. J. Fleet Spier, of Brooklyn, N. Y., being the author.

The meeting then adjourned to 4 o'clock P. M.

AFTERNOON SESSION.

At 4½ o'clock P.M., the Association was called to order, the President, Dr. Davis, in the Chair, and Dr. Furman, Secretary.

Resolutions complimenting the Committee of Arrangements, Secretary Furman, the officers of the various Institutions, and the Profession of the city, were unanimously adopted.

Dr. A. B. Palmer, also offered resolutions recognizing the services, patriotism, and sacrifices of the Medical Officers of the Army and Navy, which were adopted with unanimity and enthusiasm.

Dr. L. A. Sayre, of N. Y., moved that a Committee consisting of Drs. N. S. Davis, of Chicago, Samuel Willard, of Albany, and J. F. Hibberd, of Richmond, Ind., be appointed to revise the Constitution, Bye-Laws, and Ordinances of the Association, for the purpose of harmonizing the several provisions without introducing any new matter. The motion was adopted.

Resolutions concerning the death of Dr. B. F. Bache, of Philadelphia, were referred to the Standing Committee on Necrology.

The Secretary of the Section on Chemistry and Materia Medica, reported that the several papers referred to it, had been considered, and their reference to the Committee of Publication recommended. The report was adopted.

The Secretary of the Section on Meteorology, Epidemic Diseases, &c., &c., reported in reference to the several reports and papers referred to it, which report was adopted.

The Secretary of the Section on Surgery, reported that the several papers and cases referred to it, had been examined and discussed with much interest. The report was adopted.

The Secretary of the Section on Practical Medicine and Obstetrics, reported that the number of reports and papers referred to it, was such as to require further time to complete their consideration. Leave was given the Section to hold an evening session, and make a final report directly to the Secretary of the Association.

Dr. J. Homberger, of N. Y., offered the following:

*Whereas*, The position of Specialists and Specialties is but very ill-defined, be it

*Resolved*, That the American Medical Association go into a Committee of the Whole, in order to define the position of Specialism and Specialists, as well as the duties of the profession towards Specialists, and the duties of Specialists.

After some discussion the resolution was referred to a special Committee, consisting of Drs. J. Homberger, of N. Y., H. R. Storer, of Boston, W. Jewell, of Philadelphia, W. Hooker, of New Haven, and J. Brinsmade, of Troy; with instructions to report at the next annual meeting of the Association.

On motion of Dr. McGugin, of Iowa, a resolution was adopted recommending the passage of Laws providing adequate compensation to medical witnesses in courts of justice.

The amendment to the Constitution proposed at the last annual meeting, providing that the President and Vice-Presidents elected at one annual meeting, do not enter upon the discharge of their duties, until the commencement of the next, was called up, discussed, and adopted unanimously.

A resolution *protesting against* the appropriation, by Congress, of any reward to Dr. Morton for his pretended discovery of Anesthetics, was adopted by a very large majority.

A vote of thanks to Dr. N. S. Davis, for the able manner in which he presided over the meetings of the Association, was adopted unanimously.

On motion, the Association adjourned to meet in Boston, Mass., on the first Tuesday in June, 1865.

*Remarks.*—Thus ended one of the largest, most harmonious, and profitable meetings of the American Medical Association. The business was all conducted in good order; the reports and papers presented were numerous and valuable; the discussions in the several Sections animated and profitable; and the evening entertainments, all that liberal hospitality and high intellectual culture could make them. The whole of Friday was devoted to an excursion from Bellevue Hospital to the Institutions on Randall's and Blackwell's Islands. The day was very pleasant; the arrangements, under the admirable leadership of Dr. Taylor, excellent; and the large company in attendance reaped a rich harvest of enjoyment.

On Saturday, a large number accepted the very kind invitation of the Medical Director, and visited the Military Hospitals on David's Island, but previous engagements prevented us from enjoying their company.

---

### SEMI-ANNUAL MEETING OF THE ESCULAPIAN SOCIETY

---

*Kansas, Ill., May* 30, 1864.

PROF. N. S. DAVIS.

The Semi-annual Meeting of the Esculapian Society was held at Kansas, Ill., Wednesday, 25th inst., Dr. J. M. Steele was chosen President, and George Ringland, Secretary *pro. tem.*

A highly interesting interchange of opinion on subjects of practical interest in Medicine, Surgery, and Obstetrics, served to make the occasion pleasant and profitable to the members present.

So many of the most active members of the Society being absent in the army, it is with some difficulty that its vitality can be maintained, but a strong determination was manifested at this meeting to keep it alive till "this cruel war is over;" when we anticipate a glorious re-union of all the surviving members. I was absent a part of the time, so that I am unable to give you a synopsis of the discussions.

The Annual Meeting is to be held at Paris, on the last Wednesday in October, next.

Yours, most Respectfully,
GEORGE RINGLAND,
Secretary *pro. tem.*

---

DR. LANGER, OF DAVENPORT, ON SUB-CUTANEOUS MEDICATION.—We cheerfully bear testimony to the correctness of the statements of Dr. Langer, on the above subject, as given in another part of this number of the EXAMINER.

Book Received.—The following work has been received, but too late for extended notice in this number. It shall be examined in future:—

A Treatise on Chronic Inflammation and Displacements of the Unimpregnated Uterus.

By W. H. Byford, A.M., M.D., Professor of Obstetrics, &c., Chicago Medical College, Med. Dept. Lind University.

Philadelphia: Lindsay & Blakiston. 1864.

---

Foreign Intelligence.—Prof. Langenbeck, Surgeon-General of the Prussian Army, has been allowed to visit the wounded German prisoners in Copenhagen by the Danish Government and to return.

Rudolph Wagner, the celebrated physiologist, whose death took place in May, was appointed Professor of Zoölogy at Erlangen in 1833, but was called to Göttingen in 1840. His reputation was in a great measure made by the publication of his "Handwörterbuch der Physiologie," which, however, was only partly his own work.

The funeral of Prof. Oppolzer's wife was made the occasion of expressing the profound respect and love which is felt for this renouned teacher in Vienna. The heads of the University and all the students joined the procession, with funeral songs and torches, and the streets were thronged with sympathizing spectators.

Virchow, in the last number of his "Archiv," reports a case of trichinosis in Davenport, Iowa, as the first which has occurred in this country. The worms were discovered in the scirrhous breast of a woman after its removal, and a portion of the same was sent to him for examination. What is of chief interest in the case is the fact that the symptoms of trichina poisoning were noticed seven or eight years before the operation, and yet the worms were found alive.

Important data relating to the period of incubation of the poison of rabid dogs are given in the *Medizinische Jahrbucher*. The period was determined in 224 cases. In 40 cases less than one month; in 143 cases from one to three months; in 30 cases from three to six months; in 11 cases from six to twelve months.

The prize of 50,000 francs, offered by the Emperor Napoleon for the most useful application of electricity, has been awarded to M. Ruhenkorff for his induction coil.

Prof. Czermak, the eminent physiologist, has resigned his professorship at Pesth and returned to Prague, where he has founded a private physiological institution and laboratory, and is about to publish occasional "Mittheilungen."—*Boston Med. Journal.*

---

MEDICAL NEWS.—Mr. Edward Parish succeeds Prof. Thomas in the Chair of Materia Medica in the Phila. College of Pharmacy.——Dr. Dunlap, of Springfield, Ohio, employs permanganate of potash with great success in spotted fever, giving one-eighth to one-half a grain in solution, frequently repeated.—— The Surgeon of the Pirate Alabama was an Englishman, and his fellow students propose to erect a tablet to his memory.—— The guillotine is named from Dr. Guillotin, who proposed the law requiring that all criminals condemned to death "should be beheaded by means of a simple machine;" he did not invent the machine, as has been alleged.——Dr. Carnochan has amputated at the hip-joint five times.——Prof. Gross has in preparation a third edition of his System of Surgery, and Prof. Stille a second edition of his work on Materia Medica.——Prof. Miller, of Edinburgh, is said to have been killed by a review of his "System of Surgery." The *Times and Gaz.* says: "He was a tetotaller, and consequently did not give his brain that rest and refreshment, that power of discarding and wiping out irritating and exciting trains of thought, which wine, temperately used, will confer. Had he taken a little wine, and excited himself less, he would have written a better book, and might have laughed at reviewers."——Dr. Roberts Bartholow has resigned his commission of Assistant-Surgeon U. S. Army, and entered into private practice at Cincinnati, Ohio; his address is 344 Race Street. Dr. B. also proposes to engage in private instruction of medical students, or young men desiring to enter the army or navy.——Prof. Weber, of Cleveland, Ohio, is the President elect of the Ohio State Medical Society.—*Am. Medical Times.*

---

THE CINCHONA PLANT IN JAMAICA.—In the autum of 1860, a quantity of the seeds of several species of the Cinchona were received at Jamaica. By the month of October following, about 400 healthy plants were raised, but owing to the low level at which they were planted, more than half perished. They were then removed to an elevation of about 4000 feet above sea level, with the best results. In twelve months a plant of the red bark (Cinchona succirubra) had attained the height of 44 inches, with leaves measuring $13\frac{1}{2}$ inches in length by $8\frac{3}{4}$ inches in breadth. The culture of the Cinchona micrantha (grey bark) has not been so successful; the leaves are, however, much larger. There is abundance of land in the island possessing all the conditions requisite for the growth of the plant.

